# A Metaphysico-Physiological Essay upon the Nature of the Triple Alliance of Corporeal Organization, Animal Life, and the Life of the Understanding, as Exemplified in the Constitution of the Human Species

**Published:** 1828-11

**Authors:** William John Thomas


### PHYSIOLOGY.

### *A Metaphysico-Physiological Essay upon the Nature of the triple Alliance of Corporeal Organization, Animal Life, and the Life of the Understanding, as exemplified in the Constitution of the Human Species.* By WILLIAM JOHN THOMAS, M.R.C.S.L.

MAN is endued with volition, which is manifest from the voluntary movements performed by the body, in accordance with the several conceptions of the mind.

When death has ensued, none of the members of the body move by an impulse from within: voluntary motion has ceased.

Thought, imagination, reason, and the several passions, have also ceased; that is to say, they produce no effect upon the body: therefore, because there are no manifestations of them upon the corporate system, we conclude that they have ceased to associate therein.

Now, when we perceive a body influenced by voluntary motion, and obeying the impulses of the imagination at one time, and at another ceasing to be affected thereby, we are, of course, warranted in concluding that the thing which acted upon the living body is distinct from the body it acted upon. Because, if it were identified with that body, death, or a cessation of the manifestations of life, could not take place until a physical decomposition of the component particles of that body had ensued: in other words, until incorruption had put on corruption.

Nevertheless, the body, after the cessation of volition, not immediately corrupting, we are led to inquire into that singular phenomenon: the truth, therefore, appears to be, that there is a certain pervading principle, which gradually subsides after the separation of the faculty of volition; and this principle, which becomes weaker in proportion to the duration of time which has elapsed since the death of the body, may be called the life of the animal, in contradistinction to the faculties of reason, volition, and cogitation, which have been denominated "the life of the understanding."*

* Mr. GUTHRIE, in the physiological department of his Surgical Lectures, states that Dr. Reynolds, vicar of Kensington, first pointed out this essential diversity.

We are therefore aware that animal life, which forms a medium of materiality, and which disposes the body to be acted upon, really subsists in that body, until separated by chemical decomposition; because, if we take a human body, upon which the mental powers have ceased to operate,—if we take such a body a few hours after death, and stimulate it by galvanism, the muscular fibres immediately contract, and we find the momentum of impression to be in equal ratio with corruptibility of that body; the corporeal susceptibility being greater immediately after than at a remote period from the escape of the rational faculty.

Now, although we can restore muscular motion, we can by no means restore the volition which previously controlled it; and this I apprehend to be another proof of a diversity between the two.

Having thus briefly referred to the three great principles which enter into the constitution of mankind, viz. the material; the pseudo-immaterial, or medium called animal life; and the immaterial, cogitative, or rational faculty, called the life of the understanding; it may be expedient to consider the several purposes for which they were respectively combined.*

When it becomes necessary for an immaterial essentiality to be informed of the existence of a material universe, we may conjecture that such information might be immediate; but, since it hath pleased the omniscient Creator to confine an immaterial principle within a corporate material, what wisdom is there displayed in the construction thereof, with sentient organs, for the conveyance of impressions to the imprisoned mind!

If the organs of sensation were absent, what would be the condition of man? If the body were destitute of the organs of hearing, of sight, of taste, the tactatory implements, or olfactory organs, would life be a dream, a continued dream? The question is too complicated to be immediately resolved.†

* It is necessary to state, that, although (to avoid circumlocution and confusion) we call the faculties of reason, judgment, volition, &c. the Life of the Understanding, yet we do not consider them as such, either individually or collectively, but merely as modes of manifestation, or proper functional operations of a principle, whose essence is incomprehensible by our finite capacities. We must not, then, confound the cause with the effect. The life of the understanding may be therefore defined, that abstract principle whose existence we recognise by the several actions and passions of the mind.

† The fœtus in utero may reasonably be persumed to be possessed of sensation; and, if possessed of sensation, why not of perception? and, if it be a percipient being, it assuredly may possess innate ideas: if, therefore, it were born with an abolition of all sensation, who will presume to affirm that it might not contemplate the ideas it had formerly imagined, now preserved in the memory? and, if that were the case, what mode of existence would it then

Passages that lead to nothing

If the soul existed in the body under these conditions, it is evident that it could derive no information *ab externo*, and could by no means be cognisant of a material world, since it is separated by matter from the surrounding universe; for, if there were not sentient organs, how could the soul perceive? The body, therefore, appears to be furnished with organs of sensation for the express purpose of the soul's communication with the circumjacent world.

Moreover, that the immaterial cogitative principle might not be impeded by an immediate communication with the gross material of the body, it appears as if the life of the animal were superadded, to keep the combined functions in continual co-operation.

That the body exists but for the soul, will I trust be subsequently proved. To the mind belong all those delightful faculties, energies, and associations, which constitute the felicities of our present existence: perception, intellectual discrimination, rational decision, volition, and potential efficiency. That these are present during life, all are assured of; and, that after death they have ceased to associate in the body, none will deny.

If, therefore, these be the acknowledged faculties of the soul, exercised during life, and manifested through the mediation of the body;—if, after death, the body cease to indicate the existence of these faculties therein, and the cessation of vitality proclaim the separation of the soul from the body;—does it require an enthusiastic imagination to presume that those faculties are inseparable from the soul, and exist therein, notwithstanding the soul's separation from the body?

By no means. Enthusiasm must have no place in the calm contemplation of this sublime branch of metaphysical philosophy; for, as the secretions of the tears, the bile, the gastric and the pancreatic juices, are the proper functions of an animal body, so may the exercise of judgment, imagination, and reason, be the proper operations of the soul.

Thus we perceive that, after the death of the body, the life of the understanding may exist alone, and that, separated from the influence of the laws of matter, it ceases to be regulated thereby. Here may we indeed pause, to behold in prospect this scion of immortality. Unrestrained by the laws of gravity, the soul may ascend from earth in an instant, and perceive the universe of the creation.

enjoy? I do not here allude to the innate ideas which Mr. Locke has so logically argued against: they concerned the fundamental principles of moral truisms, which it had been presumed by some philosophers were implanted in the understanding of the fœtal being.

Trace every star, and every gem,
In night's imperial diadem.

Uninfluenced by the density of matter, how amply may her operations extend? Volition may will, and judgment shall direct those volitions. The soul has but *to will*, in the potential efficiency of its amplifying powers, that which no physical combinations, no material organization, can be permitted to withstand.

Perception may then become more percipient, reason more rational, and intelligence still more intellectual, until the magnificent schemes of the sublime Sovereign of the universe shall be fully reviewed in perception, and thoroughly understood. Amid the dark empires of materiality, in which, when conjoined to the body, this immaterial essentiality was compelled to reside, what could be more rapid than the perception manifested from within? If the eye were turned upon some brilliant ball, many millions of miles distant from this earth, how immediately was the same perceived! Can we then doubt that volition may be hereafter equally as easily executed as perception was instituted when the mind was imprisoned in the body? It is rational to presume that it may. At present, therefore, we may imagine a soul exclaiming to itself, "I will!" to perceive yon polar ball, and immediately the same is beheld. Hereafter, when the disjunction of the tri-entities has ensued, may not the same spirit of cogitation exclaim "I will!" to be seated on yon polar ball: "I will! my translation to the extremity of this celestial universe!" and immediately that volition of the mind, uninfluenced by the laws of matter, and with ten thousand times the rapidity of the transient lightning, shall be instantly executed.

These hypotheses cannot be considered either improbable or inconsistent, when it is remembered that the immateriality of the soul alone exempts it from the laws which regulate the material universe. It would be madness to will (in our present condition) our translation to a certain star at the extreme verge of creation, because during this life the soul and body are inseparably combined; and, since the soul is inseparable from the body, and the latter subject to the laws of gravity, so therefore the former is mediately affected thereby, and must necessarily be influenced through the mediation of the body by the same physical laws; and in this respect the volition of the soul, as contrary to the laws of gravity, cannot be executed.

I shall pass over the theories of life, which some of our eminent physiologists have supplied us with: the object of the present paper is to propose, and not to refute opinions.

The subject which we are now treating upon is most important: Socrates, Plata, and Pythagoras, have illuminated it by the lights of their investigations and sapient opinions; and, more recently, the immortal Addison has considered it worthy of the most profound contemplation. Posterity has already applauded his valuable writings, and crowned him with garlands of unfading bloom.

But to return to our subject. It will be easy for us now to conclude, from the foregoing observations, that the soul and the body are distinctly different, in every and all possible degrees: the body influenced by laws which cannot operate upon the soul *per se*, but which partially exercise their influence upon it, through the medium of the body: the latter, after the soul's separation therefrom, becomes subject to destructive decomposition; and why not previously? Because, when this body was united with the soul, a portion of the latter's immaterial property of incorruptibility may be presumed to have pervaded the body, or to have influenced the susceptibility of the latter.

When we look back upon the opinions of the ancients, and more especially of the Greek philosophers, on these important philosophical problems, we perceive the hypotheses I have before adverted to faintly delineated. The dialogues of Plato, and more especially some parts of the writings of Pythagoras, I might properly refer to. This philosopher seems indeed to have entertained opinions respecting the diversity of animal life and the life of the understanding: the latter he most certainly denoted by the word soul; and the former, I apprehend, he alluded to in that which he calls the luminous body, or chariot of the soul. Yet, from the manner in which he delivers his sentiments upon these subjects, we are led to conclude that he considered them to be inseparably coexistent after their dislodgement from the body, and independent of all physical and material laws.*

* Pythagoras appears to have imbibed the tenets of the Egyptians upon some particular points, (whose country, following the example of Solon and others, he had previously visited.) The Chaldeans and Egyptians held nothing to be incorporeal, or insubstantial, but the Ens Entium, who had existed *ab initio initii*. These philosophers imagined the soul to be a compound of understanding and soul: they gave the name of soul, and chariot of the soul, to the subtle body with which they conjectured the understanding was invested. Pythagoras did not acknowledge the second death of the soul, according to the Chaldean and Egyptian creeds: he maintained that there was only one death, and that that was the separation of the bi-entities of the soul,

The reasons have been before stated which induce us to maintain the diversity of animal life and the life of the understanding. The former may be considered as a medium of materiality between the immaterial essentiality of the one and the very gross materiality of the other. It may probably be disputed how any thing can be neither an immaterial essence nor a material substance, yet partake of both. The objection is certainly somewhat perplexing, but we shall endeavour to resolve it.

Throughout all nature there appear to be certain principles, which pervade even the most minute atom of the material world; for instance, electricity. Electricity may be called the animal life of the creation, that universe of innumerable worlds! and the soul of this shining association of stars, suns, and satellites, may be presumed to be the spirit of that divine Primordial which called the whole into existence. Little as we know of the operations of this supreme and adorable Being, (who is at once the centre and circumference of finite extension, and as boundless as the capacity of infinite expansion,) and still less of his vast designs, and the ultimate effects of the revolutions of sidereal worlds, this we are certainly aware of, that the proximate results of the movements of our own system have ever been productive of the utmost beneficence towards the creatures which inhabit this planet, and the more substantial works of the creation.

Considering these revolutions as the effects of infinite wisdom, perception, rational discrimination, and volition, continually operating thereon, can it be derogatory to that source of unlimited perfection to consider the whole universe as subservient to his will, even as the body of man is to the volition of his own mysterious soul?

In the human body certain movements are performed instinctively, some necessarily, others voluntarily; in the universe, faint analogies are perceptible thereto. Thus, gravitation, and the immutable physical laws of the material world, may be well accounted ordinances of the Creator, subject alone to mutability and variation from his particular volition. It is not, therefore, presumed that the ordinary and a chariot of the soul, from the body; the soul and its chariot being translated, coexistent and inseparable, to their predestined star. The Egyptians maintained the doctrine of the metampsychosis, commonly called the transmigration of souls. The Babylonians, we may presume, held the same opinions, as the remarkable case of their king Nebuchodonosor justifies us in supposing. But Plato, in his Phædon, informs us, through Socrates, that the souls of the righteous were immediately translated to the Supreme Being, and that the wicked alone were punished by a transmigration through the bodies of beasts.

operations of nâture are particular instances of his immediate interposition; but the effects of general laws, which he has especially ordained.

Animal life, which we have already instanced as a medium of materiality, between the immaterial essentiality of the soul and the materiality of the body, appears indeed to result from an organic and physical decomposition of certain principles contained within organised materials; such compositions being concordant with certain physiological ordinances which regulate those organs. It is a remarkable fact, and well deserving of particular attention, that no living body can be renovated except from the decomposition of *an organised body similar to itself.*

Now, animal life, (which that immortal ornament of our profession, JOHN HUNTER, very properly compares with electricity,) seems to subside gradually from an organised body after death, no fresh supply being generated therein; and that which had been previously elaborated is rapidly extricated after the body has ceased to live, being completely separated by the chemical decomposition of the body it previously pervaded.

I inspected the dead body of an animal, a few seconds after it had ceased to move. Upon opening the thorax, the systole and diastole of the heart were very perceptible; and, although that organ was detached from its connexions, and placed upon the table, still the alternate actions were maintained, which, by repeated touches of the scalpel, were rendered manifest for some time. The fibres having become cold and rigid, the irritable principle appeared to subside, and the motion to cease.

Thus it appears that animal life was still active in that organ, notwithstanding the apparent death of the body; and when the stimulus of the steel had ceased to impress the irritable principle, the more potent galvanic applications would have rendered the organic motivity manifest again. The food of animals, in general, is either of animal or vegetable origin. Vegetables are organised, as well as animals; they live, they circulate the nutritious particles absorbed from the air and earth; propagate their species, and die. Instinct appears to direct them in the selection of their food. The vegetable kingdom being therefore endued with so many of the vital functions common to organised bodies, may be regarded merely as a modification of the animal kingdom, and as such pervaded by the same principles of vitality which constitute the animal life of beings of superior orders.

It appears to be this principle which preserves the fluidity of the secretions in the living body. The blood may be said to possess vitality whilst circulating in its fluid state through the several sanguiferous organs; and to die, when, extra corpus, it coagulates. Does not the death or coagulation of the blood depend upon the extrication of the animal electricity, which previously pervaded it?

When either animal or vegetable matter begins to putrefy, it ceases to be proper food for man and animals. When, however, the food is received in its recent state into the stomach of animals, it loses the major part of the vital principle which had previously preserved it fresh and uncontaminated by putrefaction; the material parts being excreted, minus that proportion of the vital principle absorbed by the capillary orifices of the chyliferous absorbents, rapidly putrefies. We therefore observe that the vital principle is absorbed into the living body, and that the chyle is saturated therewith.*

We can easily perceive the route of this vital fluid, through the mesenteric glands, the receptaculum chyli, the thoracic duct, the subclavian vein, the right auricle and ventricle of the heart, the pulmonary artery, the pulmonary veins, the left auricle and ventricle; from whence it is driven to the brain, or distributed to the rest of the body.

The brain may be emphatically denominated the reservoir of animal life; for there can be little doubt that this principle is secreted off from the chyle, (now mingled with the blood,) and prepared in the brain for distribution to the body through the medium of the nerves.

I may here remark how jealous nature appears to be in the admission of doubtful food into the stomach, and into the rest of the body.

If the food be minus the vital principle, (in other words, putrefied,) the most violent nausea and vomiting ensue, until the deleterious materials be ejected.

If, notwithstanding the vigilance of nature, the contaminated food pass the pylorus, she immediately relaxes that sphincter, and, by a spasmodic contraction of the gall-bladder, (a co-relative spasm of the stomach, of necessity, taking place,†) a quantity of bile is poured into the duodenum, which facilitates the escape of the pernicious ingredients.

If, notwithstanding all these exertions, a portion only of

* The chyle may be observed to coagulate when taken out of the living body. Would continued streams of electricity delay the coagulations of the chyle and the blood, extra corpus?

† That acute observer of nature, Sir Anthony Carlisle, remarks that, whenever a spasm of the stomach takes place, a corresponding spasm of the gall-bladder ensues, and V.V.

the vitiated ingesta become agglutinated to the minute insertions of the chyliferous absorbents, seated upon those duplications of the mucous membrane of the intestines, called the valvulæ conniventes, what are the consequences? Nature (to use the words of Sir ANTHONY CARLISLE,) being aware that the vitiated chyle will be prejudicial to the system at large, fortifies the entrances by a salutary inflammation of the adjacent absorbents, thereby intercepting the inlet of the fluid. The glandulæ primi generis of the mesentery become swollen, and their calibres obstructed.

If severe vomiting and purging do not eject the morbid matter from the primæ viæ,—if nature fail, by obstructing the absorbents, to arrest all the morbid ingesta,—and a portion, only, becomes mingled with the circulation, a violent fever is the result! and, if the morbific materials be not expelled from the system by the salutary secretions and excretions, preternaturally excited, nature, being conscious that she can no longer withstand the united powers of functional derangements and corporeal disorganizations, severs that mysterious bond which binds the life of the understanding and animal life to the body: the latter dies; the life of the animal is extricated gradually from the body,—perhaps to revive some drooping flower of the desert, or, being wafted by a transient gale, it enters the grand chemical laboratory of the elements: there may it remain until nature, ever economical in the expenditure of material, employs it in her works again.

But the life of the understanding, the rational soul, now enters into life indeed! Having already delivered my own sentiments upon the nature of the future existence of the soul, I shall conclude this Essay by adducing those of philosophers (whose opinions have weight in themselves) upon the several subjects on which I have respectively treated.

PYTHAGORAS concludes his Golden Verses with the following sublime apostrophe to the soul: "Thou shalt leave the body, and mingle with the pure ether, and be immortal with the immortal gods, having abandoned mortality!"

Mr. LOCKE, in his Essay on the Human Understanding, considers that we have equally as clear ideas of immaterial as we have of material entities.

We have no idea of abstract substance, when the modes thereof are taken away; and we have no idea of the abstract essence of spirituality divested of the several operations of the mind.

Yet we do not deny the existence of matter, because we cannot deny the abstract substance thereof, since extension,* solidity, and magnitude, which characterise the presence of matter, are complex ideas, which we respectively imagine within us;" and are accustomed to imagine some *substratum* wherein they do subsist, and from which they do result, which therefore we call substance.†

This eminent philosopher opines "that we have not more or clearer primary ideas belonging to body, than we have belonging to immaterial spirit.‡

Again, "Every act of sensation, when duly considered, gives us an equal view of both parts of nature, the corporeal and spiritual; for whilst I know, by seeing or hearing, &c., that there is some corporeal being without me, the object of that sensation, I do more certainly know that there is some spiritual being within me that sees and hears. This I must be convinced cannot be the action of bare insensible matter; nor ever could be, without an immaterial thinking being."

The Cardinal Ganganelli, most eminent among the literati of the last century, in a letter addressed to the Abbé Genovesi,§ thus speaks of the progressive development of the intellectual faculties: "If the soul, like a budding flower, expands by insensible degrees, the reason is, that it depends upon a body sluggish in its progressions." Again,|| "Man quits the paths of reason when he attributes those astonishing operations to the inert mass of his body, and dares to attribute the honour of them to the acrimony of his bile, or the quick circulation of his blood: none but a spiritual being can produce immaterial ideas. The most subtle particles of air might be collected, might be agitated in every direction, but never be formed into a syllogism. Flame, radiant and penetrating as it is, has never yet given birth to a single thought or a single argument. That thought, which in an

* These complex ideas may, by abstraction, be analysed into separate simple ideas: we cannot have an uncompounded idea of extension, because we must have the idea of substance extended; and substance cannot be extended without displacing space, equivalent to the bulk of the parts extended. Neither can a body be extended without form and magnitude, which comprehend all the mathematical varieties of configuration. Therefore, when extension is comprehended in contemplation, we necessarily have the ideas of extent, space, figure, colour, and a thousand other co-relative, complex, and simple ideas, connected therewith. We cannot form an idea of solidity without resistance; or resistance, without matter; or matter, without magnitude and form; and who can imagine the idea of magnitude, without finite extension, or infinite expansion?

† Locke on Human Understanding, chap. 23, § 1.

‡ Ibid. chap. 23, § 16.

§ Vide Letter cxxxvii. || Ibid.

instant makes the circuit of the world, which subjects the universe to its observation, which with the most rapid flight rises even to the Infinite Being,—which hath neither situation, figure, nor colour,—which imperiously commands, and forces the body to obey its orders. Tell me how it can be part of the same body?"

In concluding this Essay, I beg leave to observe, that the doctrine of the materiality and mortality of the soul should for ever be exploded as totally false, and unworthy of all regard,—as subverting the fundamental principles of all religions,—as introducing civil anarchy into the political economy of legislation,—as substituting discord for harmony, despair for hope, and eternal darkness for everlasting light.

Truth! how many dangerous and demoralising doctrines are promulgated in thy name!

*Liverpool; October* 1828.

